# Supplementary material for: Earth Worming—An Evaluation of Earthworm (Eisenia andrei) as an Alternative Food Source
Source: Foods. 2023 May 10;12(10):1948. doi: 10.3390/foods12101948 (PMC10217592; doi:10.3390/foods12101948)
Supplement: Supplementary file 1 [file foods-12-01948-s001.zip › foods-2350424-supplementary.pdf]

Earth Worming— An Evaluation of Earthworm (*Eisenia andrei*) as an Alternative Food Source

Supplementary Materials

Table S1. The protein yield and purity of EAPPE was expressed in % DW.

	Yield	Purity
**EAPPE**	22.1 0.27	78.7 0.71
